# Put St. George's Hospital First

**Published:** 1914-02-07

**Authors:** 


### PUT ST. GEORGE'S HOSPITAL FIRST.

### THE PAST, THE PRESENT, AND THE FUTURE.

Not a little confusion has been created in the minds of the public, the profession, and the governors as to what are the real issues raised by the persistent attempts to provide one of the treasurers with a paid appointment in connection with St. George's Hospital. Unfortunately, as not infrequently occurs where personal considerations are involved, the safeguarding of the interests of a great hospital like St. George's seems to have been sometimes suspended or forgotten altogether in the controversy which has arisen in consequence. It was a mistake for Mr. West to allow himself to be nominated for the post of paid treasurer, which involved a total change in the system of administration which has prevailed at this hospital since it was first established. The days of paid treasurers have long since ended in this country, and this change has been beneficial to the government of our voluntary hospitals. On the defeat of this proposal, which was disposed of finally by the House Committee without one hand being raised in support of it, it was most unfortunate that Mr. West and his friends should have proceeded to a course of action which left St. George's Hospital without a medical superintendent for a period, against the declared wishes and advice of the medical staff committee and the strong protests and sound reasoning of most of the lay members of the House Committee. The affairs of a big hospital cannot be efficiently administered under such circumstances, and St. George's Hospital may have suffered in consequence.

### Influences and Causes.

It would have been well for St. George's Hospital and for everyone concerned if the defeat by the governors of the proposal to provide Mr. West with a paid post had been accepted by all parties, and the New Year had been begun with all the governors uniting to put their shoulders to the wheel and join hands in a determined effort to remedy the mistakes of the past and put the interests of St. George's Hospital before all other considerations. Under the laws the decision of the Special Court on December 19, 1913, was final and could not be re-opened. Instead of recognising this position, the controversy was kept open by influences and causes which may be made plainer by the proceedings at the Special Court to be held on the 12th instant. It was first announced in the *Times* that the President had given directions for " an independent inquiry into the charges brought by certain governors at the meeting on December 19 against the administration of the hospital by the treasurers." This was an unfortunately worded mandate, for, as we pointed out in our issue of the 10th ultimo, no charges of any kind were brought by certain governors at the Court in question against the administration of the hospital by the treasurers. It has not been stated yet who it was who advised the President in this matter, and who is responsible for the wording of the mandate in question. The President has acted in good faith throughout, and seems to have been given to believe it to be the wish of the House Committee that this so-called independent inquiry should be held at her instance. Her Royal Highness was, therefore, put in a false position when it was made plain to her, subsequently, that some of the parties to the dispute would not accept as final the findings of the commission if appointed.

### A Surprise for the House Committee.

This regrettable position came as a surprise to several members of the House Committee at their meeting on the 28th ultimo, and they very properly decided to approach Her Royal Highness to ask for an interview to enable them to make all the reparation in their power to the President for what had happened. Her Royal Highness in the exercise of her discretion has not seen her way to accede to this request of the House Committee, though she has thanked them for the kindly feeling which prompted the suggestion, but has decided to adhere to her resignation. The House Committee wished that Lord Plymouth should be invited to remain one of the treasurers, seeing that he has had nothing whatever to do in any way with the matters in dispute, nor was he mentioned or referred to by any speaker or at all at the special meeting of governors on December 19 last. Lord Plymouth has, however, determined to retire from the treasurership. At a meeting of the House Committee on the 4th instant a committee was therefore elected to appoint two treasurers in succession to Lord Plymouth and Mr. A. W. West. We are glad that notice has been given of " a resolution of thanks to the retiring treasurers for their services to the hospital, and that they be asked to accept the position of honorary life governors." We have no doubt that this resolution will be unanimously agreed to.

### Some Matters for Regret.

There is one matter which we consider very regrettable, and that is that practically throughout the whole period from June 1913 Mr. A. W. West has remained in entire charge of the administration of St. George's Hospital and in a position of supreme control. It has fallen to his lot to settle all the minutes of the proceedings which are to be found in the minute-book of the hospital during this period, and in the ordinary course to issue the official notices sent to the press, and to communicate with the latter in regard to the matters in dispute and the state of affairs from time to time. It would have been better and more in the interests of peace and of St. George's Hospital itself had everyone personally interested in the results of the proceedings in question followed the equitable and wise course of holding entirely aloof from the active administration and control of the business of St. George's Hospital during this period. Had this usual procedure been followed we believe that long ago the whole of these matters could have been happily settled, and some things which cannot tend to promote the well-being of St. George's Hospital might have been prevented. It is unfortunate, for instance, that the *Times* of January 29 should contain a statement that "the treasurers have been asked by the House Committee to hold over their resignations until after February 12, but that Mr. West has declined to accede to this request," seeing that, in fact, no such request was made to Mr. West by the House Committee.

### The Policy Experience Justifies.

We are sincerely anxious that the meeting of the governors on the 12th instant shall finally end these unfortunate disputes, that the election of new treasurers, the filling up of the vacancies which have occurred on the House Committee, and the appointment of a committee of five or seven governors to consider the laws of St. George's Hospital and report to a Special Court as early as possible if any and what alterations should be made therein, shall be unanimously approved by the governors. By thus sticking closely to business the governors will be able to re-establish peace, and to place the interests of St. George's Hospital once again in the position of being paramount, which they ought always to have held. These personal questions have been debated and raised to an importance which they ought never to have been allowed to occupy at all, and much less for many months. The governors will, we hope, set their face against any re-opening of these personal matters in any form or shape at the meeting on the 12th instant. The governors may so secure that the ordinary and pressing business of the hospital shall be taken vigorously in hand and pursued without interruption until the amalgamation with Westminster Hospital is completed, the new laws and regulations are ready for adoption, and the whole of the business of the hospital has been brought into the highest state of efficiency. All these things, and the immediate reorganisation of the financial administration of the affairs of St. George's Hospital, ought to be capable of being successfully accomplished by the date of the meeting of the Court of Governors in June next. At that Court, a new House Committee will have to be elected in the ordinary course, and thenceforward St. George's Hospital, strengthened and invigorated, should be put in a position to become one of the greatest and most successful voluntary hospitals in the Metropolis.

### A Word of Explanation.

The matter we referred to earlier is one that has properly caused serious annoyance to members of the acting medical staff especially. At the meeting on December 19 one governor used the words "medical school" instead of "medical staff," and another made certain statements which he could not have carefully verified beforehand. The latter speaker read from a manuscript and could only be heard by a few of the governors who were near him, and then but indistinctly. His written notes, however, were published in full in some papers, and have created a great deal of feeling. That feeling is to find expression at the meeting of the Court on the 12th instant, when amongst the special business appears the following: "To consider and, if approved, to move a resolution regretting the action of certain governors, who, after bringing charges against Mr. West, as treasurer, and the medical staff, declined to abide by the result of an independent inquiry which the President proposed to institute, and which the House Committee had welcomed." We gather from the wording of this resolution that the "independent inquiry" was suggested not, as many supposed, to endeavour once more to find Mr. West a paid position at St. George's Hospital, but to deal with the published statements made by the two speakers referred to. These speeches, in the circumstances, were felt not to be of sufficient importance to move the President to direct such an inquiry, but the above resolution, it will be observed, covers wider grounds, for it includes the action of the governors who declined to abide by the findings of the proposed inquiry should Her Royal Highness proceed with it. We hope, therefore, that any discussion which may arise on this resolution will dispose of all the points at issue and restore harmony. The *bona fides* of the disputants is not in question. So a frank admission of error on the part of any who may have fallen into it, combined with ample apology to the medical staff or to any who may be found entitled to it, should happily end the affair to the credit and satisfaction of all concerned.

### An Impartial Review of the Whole Matter.

Governors who wish to understand the matters in dispute can do so by referring to The Hospital for December 27, 1913, pages 337 to 340; The Hospital for January 10, 1914, pages 391 to 393; The Hospital for January 17, pages 419, 420; The Hospital for January 24, page 451; The Hospital for January 31, page 477. A perusal of these impartial and clear statements will place any reader in full possession of the material facts. Let every governor—and we hope all who can do so will not fail to be present—attend the meeting at St. George's Hospital on the 12th inst., at 3 p.m., fully resolved to use his influence to secure that an end shall be put to all personal questions. Further, that steps shall be taken to secure that the best interests of St. George's Hospital shall henceforth be the first and paramount qualification for all who may be entrusted with the administration of its affairs. Providing harmony and good feeling are conspicuous at the Court on the 12th inst., as we have reason to hope they may be, St. George's Hospital may thenceforward go on with its proper business to assured success, with the sympathy and increasing support of the public behind it.

